# Depressive and anxiety symptoms, quality of sleep, and coping during the 2019 coronavirus disease pandemic in general population in Kashmir

**DOI:** 10.1186/s43045-020-00069-2

**Published:** 2020-11-04

**Authors:** Bilal Ahmad Bhat, Rouf Ahmad Mir, Arshad Hussain, Iqra Rasheed Shah

**Affiliations:** Department of Psychiatry, GMC Srinagar, Jammu and Kashmir, India

**Keywords:** Coronavirus disease, Depressive symptoms, Anxiety symptoms, Quality of sleep, Coping

## Abstract

**Background:**

With uncertainty surrounding the 2019 coronavirus disease pandemic, there is no knowledge of the psychological impact of this pandemic on the general public from Kashmir. We aimed to understand the psychological impact in the form of depressive symptoms, anxiety symptoms, quality of sleep, and coping during this pandemic.

**Methods:**

This cross-sectional study was conducted using social networking sites. The questionnaire meant for this study was sent as a link to a respondent. Initial part of questionnaire collected the socio-demographic details of the respondents. Depressive and anxiety symptoms were assessed using Hospital Anxiety and Depression Scale. The Pittsburgh Sleep Quality Index (PSQI) was used to assess the quality of sleep. There was also an open-ended question to look for coping skills used.

**Results:**

The majority of our respondents were below 45 years (around 95%) with 54.9% from 18 to 30 years age group. 72.3% were males and 27.7% were females. 58.7% were from rural background. 55.7% were employed, and 32.2% were students. In our respondents, 55% had anxiety symptoms, 55% had depressive symptoms, around 53% had poor quality of sleep, and around 30% of used maladaptive coping skills. Significant depressive symptoms were there in the younger age group, 18–30 years (*p* = 0.03). Significant depressive symptoms and anxiety symptoms were present in females (*p* = 0.01 and 0.006, respectively). In urban population, significant anxiety symptoms (*p* = 0.03) were present. The mean score for anxiety symptoms and depressive symptoms was 8.05 ± 4.53 and 8.07 ± 4.56, respectively. Mean global PSQI score was 6.90 ± 3.82 and was positively correlated with score on depressive symptom scale (*p* = 0.001) as well as score on anxiety symptom scale (*p* = 0.001).

**Conclusion:**

Younger people, females, those living in urban conditions, and those using maladaptive coping skills are likely to have anxiety symptoms and depressive symptoms as well as poor quality of sleep.

## Background

The 2019 coronavirus disease (COVID-19), an infectious disease due to a newly discovered coronavirus, can present with mild to moderate respiratory illness with recovery in most people without requiring special treatment [1]. However, it resulted in an outbreak of viral pneumonia in China [2–4]. On 11 March 2020, WHO characterized COVID-19 as a pandemic, and as of now on 28 August 2020, there are more than 24 million confirmed cases and over 800 thousand confirmed deaths worldwide [5]. Since the 2003 outbreak of viral pneumonia due to severe acute respiratory syndrome (SARS), it is the largest atypical pneumonia outbreak [6, 7]. In India, on 28 August 2020, there are more than 7 lakh active cases with more than 60 thousand confirmed deaths [8]. With its first case reported on 18 March 2020, on 28 August 2020, there are currently more than 35 thousand confirmed cases with around 700 deaths till now in Jammu and Kashmir [9]. With every passing day, the number of positive cases of this disease is increasing. It is inducing fear, and there is an urgent need for understanding its psychological impact on the general public [10]. During outbreaks of infections in the past, earlier research has shown a range of impacts on the mental health of people at the individual and community levels. The negative emotions are further compounded by lockdowns, closure of business and schools, etc. [11]. During an outbreak of one of influenza, the percentage of respondents who were very worried or fairly worried about contracting the influenza virus fluctuated between 10 and 30% but in the same study, around 60% of respondents accepted the flu vaccine for fear of the contracting virus, either to themselves or to their children [12]. During this pandemic, to support mental health and to provide psychosocial support, WHO developed a series of different messages to communicate with different target groups including the general public [13]. In China, the National Health Commission there released a notification regarding the basic principles for emergency psychological interventions and care for mental health in different target groups including the general public who are in need [10]. With uncertainty surrounding this pandemic, we hypothesize that there will be a wide range of negative impacts on the psychological health of people at individual and community levels. There is limited research from India, on how situations like this affect mental health and sleep in the general public. Further, there is no knowledge of the psychological impact of this pandemic on the general public from Kashmir. Therefore, to explore this gap in research, using a social media-based survey, we tried to understand the impact on psychological health, in the form of depressive and anxiety symptoms, quality of sleep, and use of coping skills among the general public during the initial stages of this pandemic in Kashmir.

## Methods

### Study design

To maintain social distancing and prevent the spread of COVID-19, this cross-sectional survey was conducted using social networking sites.

### Participants and procedure

After getting approval from the institutional ethics committee, the questionnaire meant for this study was sent as a link to a respondent. Non-probability convenience sampling technique was used to approach the Kashmiri population on different social networking sites. On opening this link, before the questionnaire starts the nature and purpose of this survey was explained in an easily understandable language. To ensure participation of the Kashmiri population only, it was made clear that the survey is being carried out on the Kashmiri population only. This was followed by a compulsory checking of a statement of giving or not giving consent for participation in this survey. The questionnaire opens only to those participants who checked the statement of giving consent for this survey. To recruit more respondents, they were given a choice to forward the questionnaire to others on their will (Snowball sampling). Those respondents whose response to the questionnaire was incomplete were excluded from this study. The respondents who responded in affirmation to a question asking about the presence of psychiatric morbidity before this pandemic were also excluded. The survey started on 4 April 2020 and was closed on 10 April 2020 when there was no response in the last 48 h. The sample size was calculated using Cochran formula, as follows: $n=\frac{Z^2\ P\ \left(1-P\right)}{d^2}$ where *n* = minimum sample size required; *Z* = standard normal variable, which is 1.96 at 95% confidence interval; *p* is the estimated proportion of problem in population which was taken as 20%; and *d* = acceptable margin of error, which is considered as 0.05 at 95% confidence interval. Accordingly, we estimated a minimum sample size of 246.

### Tools

In its initial part, the questionnaire collected the demographic details of our respondents. To assess depressive symptoms and anxiety symptoms, we used the Hospital Anxiety and Depression Scale (HADS). This scale is a self-report questionnaire that takes only 2–5 min to complete and is comprised of seven questions each for anxiety and depressive symptoms [14]. Cut-off scores are available for quantification, a score less than seven indicates non-cases for both anxiety and depression scales, whereas 8–10, 11–14, and 15–21 scores indicate mild, moderate, and severe cases, respectively, on both these subscales [15]. HADS performed well in assessing the severity of symptoms as well as the caseness of anxiety disorders and depression in the general population [16]. To assess the quality of sleep in our respondents, the Pittsburgh Sleep Quality Index (PSQI) was used. It contains seven components with a score ranging from 0 to 3 for each component. Accordingly, the global score on PSQI ranges from 0 to 21 [17]. The higher a global PSQI score, the more sever is a sleep disorder. Manzar et al. recommended a cut-off score of > 6 for the Indian population with a global score of 7 or more on PSQI indicating poor quality of sleep [18]. There was also an open-ended question asking respondents “how are you coping with stress during this COVID-19 pandemic?” to look for coping skills used. The responses to this question were evaluated by consultant psychiatrist and were classified either as adaptive coping skills or as maladaptive coping skills.

### Statistical analysis

Statistical Package for Social Sciences (SPSS version 19; IBM Software, Armonk, NY) was used to analyze data. Qualitative data were expressed as frequencies and percentages whereas quantitative data were expressed as mean and standard deviation. To find differences in the prevalence of depressive symptoms, anxiety symptoms, quality of sleep, and coping skills among various socio-demographic variables, the chi-square test, and Fisher’s exact test were used. To compare scores for anxiety and depressive symptoms as well as global PSQI score among socio-demographic variables and the use of coping skills, the Mann-Whitney *U* test, and Kruskal-Wallis *H* test were used. In the case where the *p* value for the Kruskal-Wallis *H* test was < 0.05, further group differences were obtained with the help of post hoc Bonferroni correction (group-wise comparisons) to identify the source of the difference. Pearson’s correlation coefficient was obtained to find the relationship between the global PSQI score and anxiety and depressive symptoms score. Significance was set at a *p* value of < 0.05.

## Results

We received a total of 309 responses out of which 45 were excluded either for incomplete response or presence of psychiatric morbidity before the pandemic. As such, 264 complete responses were included for this study. The mean age of our respondents was 31.45 ± 8.48 with a range of 19–63. Table 1 depicts the socio-demographic and other variables of our respondents. As can be seen, around 55% of our respondents have anxiety symptoms. Mild anxiety symptoms were present in 68 (25.8%), Moderate in 56 (21.2%), and severe symptoms in 19 (7.2%) of our respondents. Depressive symptoms were also present in around 55% with mild symptoms in 61 (23.1%), moderate in 69 (26.1%), and severe depressive symptoms in 14 (5.3%). Around 53% had poor quality of sleep and around 30% of our respondents used maladaptive coping skills.

**Table 1:** Socio-demographic and other variables (*N* = 264)

Variable	*N* (%)
**Age**	
18–30 years	145 (54.9%)
31–45 years	110 (41.7%)
46–60 years	7 (2.7%)
> 60 years	2 (0.8%)
**Gender**	
Male	191 (72.3%)
Female	73 (27.7%)
**Domicile**	
Rural	155 (58.7%)
Urban	109 (41.3%)
**Occupation**	
Employed	147 (55.7%)
Student	85 (32.2%)
Skilled worker	5 (1.9%)
Laborer	1 (0.4%)
Businessman	24 (9.1%)
Others	2 (0.8%)
**Anxiety Symptoms**	
Absent	121 (45.8%)
Present	143 (54.2%)
**Depressive Symptoms**	
Absent	120 (45.5%)
Present	144 (54.5%)
**Quality of Sleep**	
Good	125 (47.3%)
Poor	139 (52.7%)
**Coping**	
Adaptive	180 (68.2%)
Maladaptive	84 (31.8%)

Tables 2, 3, and 4, respectively, depict the prevalence of anxiety symptoms, depressive symptoms, and prevalence of good or poor quality of sleep in relation to socio-demographic variables and type of coping skills. There was a significant relation of anxiety symptoms with female gender (*p* = 0.006) and urban residence (*p* = 0.03), and of depressive symptoms with younger age group (18–30 years, *p* = 0.03) and female gender (*p* = 0.01) whereas there was no significant relationship between the socio-demographic variables and quality of sleep. The use of maladaptive coping skills was significantly associated with the presence of anxiety (*p* = 0.001) and depressive (*p* = 0.008) symptoms, and poor sleep quality (*p* = 0.001).

**Table 2:** Socio-demographic variables in relation to anxiety symptoms (*N* = 264)

Variable	Anxiety symptoms		Total, ***n*** (%)	Fisher’s exact test/chi-square; ***p***
	Absent, ***n*** (%)	Present, ***n*** (%)		
**Age**				
**18–30 years**	60 (41.4%)	85 (58.6%)	145 (100.0%)	Fisher = 4.65; *p* = 0.17
**31–45 years**	58 (52.7%)	52 (47.3%)	110 (100.0%)	
**46–60 years**	3 (42.9%)	4 (57.1%)	7 (100.0%)	
**> 60 years**	0 (.0%)	2 (100.0%)	2 (100.0%)	
**Total**	121 (45.8%)	143 (54.2%)	264 (100.0%)	
**Gender**				
**Male**	98 (51.3%)	93 (48.7%)	191 (100.0%)	*χ*^2^ = 8.34; *p* = 0.006^*^
**Female**	23 (31.5%)	50 (68.5%)	73 (100.0%)	
**Total**	121 (45.8%)	143 (54.2%)	264 (100.0%)	
**Domicile**				
Rural	80 (51.6%)	75 (48.4%)	155 (100.0%)	*χ*^2^ = 5.05; *p* = 0.03^*^
Urban	41 (37.6%)	68 (62.4%)	109 (100.0%)	
Total	121 (45.8%)	143 (54.2%)	264 (100.0%)	
**Occupation**				
Employed	71 (48.3%)	76 (51.7%)	147 (100.0%)	Fisher = 6.28; *p* = 0.23
Student	38 (44.7%)	47 (55.3%)	85 (100.0%)	
Skilled worker	2 (40.0%)	3 (60.0%)	5 (100.0%)	
Laborer	1 (100.0%)	0 (.0%)	1 (100.0%)	
Businessman	7 (29.2%)	17 (70.8%)	24 (100.0%)	
Others	2 (100.0%)	0 (.0%)	2 (100.0%)	
Total	121 (45.8%)	143 (54.2%)	264 (100.0%)	
**Coping**				
Adaptive	98 (54.4%)	82 (45.6%)	180 (100.0%)	*χ*^2^ = 16.90; *p* = 0.001*
Maladaptive	23 (27.4%)	61 (72.6%)	84 (100.0%)	

*Statistically significant

**Table 3:** Socio-demographic variables in relation to Depressive Symptoms (*N* = 264)

Variable	Depressive symptoms		Total, ***n*** (%)	Fisher’s exact test/chi-square; ***p***
	Absent, ***n*** (%)	Present, ***n*** (%)		
**Age**				
**18–30 years**	56 (38.6%)	89 (61.4%)	145 (100.0%)	Fisher = 8.10; *p* = 0.03^*^
**31–45 years**	60 (54.5%)	50 (45.5%)	110 (100.0%)	
**46–60 years**	4 (57.1%)	3 (42.9%)	7 (100.0%)	
**> 60 years**	0 (.0%)	2 (100.0%)	2 (100.0%)	
**Total**	120 (45.5%)	144 (54.5%)	264 (100.0%)	
**Gender**				
**Male**	96 (50.3%)	95 (49.7%)	191 (100.0%)	*χ*^2^ = 6.44; *p* = 0.01^*^
**Female**	24 (32.9%)	49 (67.1%)	73 (100.0%)	
**Total**	120 (45.5%)	144 (54.5%)	264 (100.0%)	
**Domicile**				
Rural	76 (49.0%)	79 (51.0%)	155 (100.0%)	*χ*^2^ = 1.94; *p* = 0.17
Urban	44 (40.4%)	65 (59.6%)	109 (100.0%)	
Total	120 (45.5%)	144 (54.5%)	264 (100.0%)	
**Occupation**				
Employed	69 (46.9%)	78 (53.1%)	147 (100.0%)	Fisher = 4.70; *p* = 0.44
Student	36 (42.4%)	49 (57.6%)	85 (100.0%)	
Skilled worker	3 (60.0%)	2 (40.0%)	5 (100.0%)	
Laborer	1 (100.0%)	0 (.0%)	1 (100.0%)	
Businessman	9 (37.5%)	15 (62.5%)	24 (100.0%)	
Others	2 (100.0%)	0 (.0%)	2 (100.0%)	
Total	120 (45.5%)	144 (54.5%)	264 (100.0%)	
**Coping**				
Adaptive	92 (51.1%)	88 (48.9%)	180 (100.0%)	*χ*^2^ = 7.30; *p* = 0.008*
Maladaptive	28 (33.3%)	56 (66.7%)	84 (100.0%)	

*Statistically significant

**Table 4:** Socio-demographic variables in relation to quality of sleep (*N* = 264)

Variable	Quality of Sleep		Total, ***n*** (%)	Fisher’s exact test/chi-square; ***p***
	Good, ***n*** (%)	Poor, ***n*** (%)		
**Age**				
**18–30 years**	69 (47.6%)	76 (52.4%)	145 (100.0%)	Fisher = 3.30; *p* = 0.33
**31–45 years**	54 (49.1%)	56 (50.9%)	110 (100.0%)	
**46–60 years**	1 (14.3%)	6 (85.7%)	7 (100.0%)	
**> 60 years**	1 (50.0%)	1 (50.0%)	2 (100.0%)	
**Total**	125 (47.3%)	139 (52.7%)	264 (100.0%)	
**Gender**				
**Male**	96 (50.3%)	95 (49.7%)	191 (100.0%)	*χ*^2^ = 2.35; *p* = 0.13
**Female**	29 (39.7%)	44 (60.3%)	73 (100.0%)	
**Total**	125 (47.3%)	139 (52.7%)	264 (100.0%)	
**Domicile**				
Rural	78 (50.3%)	77 (49.7%)	155 (100.0%)	*χ*^2^ = 1.33; *p* = 0.26
Urban	47 (43.1%)	62 (56.9%)	109 (100.0%)	
Total	125 (47.3%)	139 (52.7%)	264 (100.0%)	
**Occupation**				
Employed	66 (44.9%)	81 (55.1%)	147 (100.0%)	Fisher = 4.31; *p* = 0.51
Student	43 (50.6%)	42 (49.4%)	85 (100.0%)	
Skilled worker	3 (60.0%)	2 (40.0%)	5 (100.0%)	
Laborer	1 (100.0%)	0 (.0%)	1 (100.0%)	
Businessman	10 (41.7%)	14 (58.3%)	24 (100.0%)	
Others	2 (100.0%)	0 (.0%)	2 (100.0%)	
Total	125 (47.3%)	139 (52.7%)	264 (100.0%)	
**Coping**				
Adaptive	101 (56.1%)	79 (43.9%)	180 (100.0%)	*χ*2 = 17.42; *p* = 0.001*
Maladaptive	24 (28.6%)	60 (71.4%)	84 (100.0%)	

*Statistically significant

The mean score for anxiety symptoms on HADS was 8.05 ± 4.53 (range 0–21) and for depressive symptoms was 8.07 ± 4.56 with a range of 0–20. The mean global PSQI score was 6.90 ± 3.82 with a range of 0–21. Table 5 depicts a comparison of scores for anxiety and depressive symptoms as well as global PSQI score among socio-demographic variables and coping using the Mann-Whitney *U* test and Kruskal-Wallis *H* test. A significant difference was found by the Kruskal-Wallis *H* test in mean ranks of anxiety (*p* = 0.04) and depressive (*p* = 0.02) scores among different age groups. However, on post hoc analysis, the 18–30 years age group as well as > 60 years age group had significantly high mean rank for anxiety symptoms in comparison to 31–45 years age group (*p* = 0.022 and 0.031, respectively). Similarly, on post hoc analysis, 18–30 years of age group as well as > 60 years age group had significantly high mean rank for depressive symptoms in comparison to 31–45 years age group (*p* = 0.034 and 0.019, respectively). Mann-Whitney *U* test showed females having a significant high mean rank for anxiety (*p* = 0.001) and depressive (*p* = 0.02) scores as well as the global PSQI score (*p* = 0.02). A significant high mean rank for anxiety score (*p* = 0.01) was also shown by the Mann-Whitney *U* test in the urban population. Significant high mean ranks for anxiety (*p* = 0.001) and depressive scores (*p* = 0.001) as well as the Global PSQI score (*p* = 0.001) were present in those using maladaptive coping skills. Global PSQI score was positively correlated with scores depressive symptoms scale (*r* = 0.56; *p* = 0.001) and anxiety symptoms scale (*r* = 0.52; *p* = 0.001) on HADS.

**Table 5:** Comparison of anxiety and depressive symptoms and quality of sleep by mean ranks (*N* = 264)

Variable	***N*** (%)	Anxiety score, mean rank	Depression score, mean rank	Global PSQI score, mean rank
**Age**				
18–30 years	145 (54.9%)	142.05	141.68	138.84
31–45 years	110 (41.7%)	118.29	118.57	121.23
46–60 years	7 (2.7%)	136.29	131.64	168.93
> 60 years	2 (0.8%)	208.75	236.25	165.25
		*χ*^2^ = 8.13; *p* = 0.04^*^	*χ*^2^ = 9.49; *p* = 0.02^*^	*χ*^2^ = 5.40; *p* = 0.14
**Gender**				
Male	191 (72.3%)	122.38	125.65	125.74
Female	73 (27.7%)	158.97	150.42	150.20
		*Z* = − 3.49;*p* = 0.001^*^	*Z* = − 2.36; *p* = .02^*^	*Z* = − 2.37; *p* = .02^*^
**Domicile**				
Rural	155 (58.7%)	121.92	127.58	127.21
Urban	109 (41.3%)	147.55	139.49	140.02
		*Z* = − 2.69; *p* = 0.01^*^	*Z* = − 1.25; *p* = 0.21	*Z* = − 1.35; *p* = 0.19
**Occupation**				
Employed	147 (55.7%)	128.96	127.97	128.84
Student	85 (32.2%)	138.45	137.91	139.34
Skilled worker	5 (1.9%)	126.40	120.90	132.40
Laborer	1 (0.4%)	13.50	5.00	41.50
Businessman	24 (9.1%)	147.29	154.96	142.88
Others	2 (0.8%)	37.25	59.25	32.25
		*χ*^2^ = 7.34; *p* = 0.20	*χ*^2^ = 7.80; *p* = 0.17	*χ*^2^ = 6.38; *p* = 0.27
**Coping**				
Adaptive	180 (68.2%)	117.51	120.83	118.18
Maladaptive	84 (31.8%)	164.61	157.51	163.18
		*Z* = − 4.68; *p* = 0.001^*^	*Z* = − 3.64; *p* = 0.001^*^	*Z* = − 4.48; *p* = 0.001^*^

*PSQI* Pittsburgh Sleep Quality Index

*Statistically significant

## Discussion

Anxiety and depressive symptoms were highly prevalent in our study with more than half of our respondents having depressive symptoms and more than half having anxiety symptoms. As mentioned early in methodology, HADS has been found to assess the caseness of major depressive disorder and anxiety disorders quite well above the cut-off value of seven. Therefore, it is alarming that more than half of our respondents qualified above this cut-off score for both these symptoms. However, the majority of those with these symptoms had mild grades of symptoms only. Moderate or severe symptoms were present in around 30% of our respondents. In 2015, a mental health survey [19] was undertaken in 10 district of Kashmir Valley to estimate prevalence of mental health-related conditions in the general public. In this survey, on screening the general public in the Kashmir Valley, approximately 26% were exhibiting signs of a probable anxiety-related disorder whereas the proportion of the adult population suffering from symptoms of probable depression was 41% [19]. Although signs and symptoms of anxiety and depression were highly prevalent, the people who met DSM-IV diagnostic criteria for severe depression were 10% only [19]. Roy et al. [20] in a study on the Indian population reported high levels of anxiety in their respondents and suggested addressing the psychological issues of people and intensifying the awareness programs during this COVID-19 pandemic. Similarly, Chakraborty and Chatterjee [21] from West Bengal found about 70% of their respondents with high levels of anxiety, and about 25% of their respondents depressed. A survey in China [7] during the initial phase of this outbreak of COVID-19 found moderate to severe psychological impact rated by 54% of respondents and moderate to severe anxiety and depressive symptoms being reported by 29% and 16% of respondents, respectively. Another web-based survey in China [22] during this epidemic found that generalized anxiety symptoms were present in 35% of respondents and depressive symptoms were present in 18% of respondents. The psychological reaction of people during an outbreak of infectious disease plays an important role in determining both the spread of the infection as well as the occurrence of psychological distress during and after the outbreak. Having said that, sufficient resources are rarely allocated to mitigate or manage the mental health effects of a pandemic [23, 24]. This could be understood during the initial phase of an infectious outbreak when priorities are given to testing, transmission reduction, and intensive care of critical patients, but it is unwise to overlook the psychological and the psychiatric needs of people during any phase of an outbreak. Disasters, whether traumatic, natural or environmental are almost always accompanied by an increase in depressive disorder, anxiety disorders, posttraumatic stress disorder (PTSD), substance use disorder, child abuse, etc. [25]. In a study from Hong Kong during the 2003 SARS epidemic [26], a high percentage of respondents were horrified, felt helpless, and were apprehensive about themselves or their family members for contracting the virus, and around 50% of them perceived that their psychological health deteriorated moderately or severely because of the epidemic. With an enhanced connectedness and increased air travel throughout the globe, the spread of this pandemic is much more effortless, and thus, compared to the 2003 SARS epidemic, the psychological fear in the current pandemic is perhaps more intensified [27]. Although a pivotal tool in encouraging people to take precautions and preventive measures, extensive coverage of the pandemic by media can influence and amplify the apprehension in the general public [28, 29]. In Canadian adults, an Angus Reid poll conducted in February 2020 [30] indicated a significant impact of COVID-19 on psychological health and about one third of respondents were apprehensive about this viral infection and 7% were very concerned about getting infected. Females comprised 27.7% of our study population and our data suggest that they are more likely to have depressive and anxiety symptoms in comparison to males and had significantly higher mean scores for these symptoms. Women have been found at higher risk of depression in epidemiological studies as well [31]. In a 2015 Kashmir mental health survey [19], being a woman was a significant predictor of mental health problems. Although the abovementioned Indian studies did not look into the gender differences of psychological impact during COVID-19, a recent study from China [7] found higher levels of stress, anxiety symptoms, and depression in females. However, another web-based survey from China did not report this gender-related difference in psychological impact [22]. In our study, the urban population reported significant anxiety symptoms in comparison to the rural population. Though different occupational groups did not show any significant difference in psychological symptoms, the mean score in students was high in comparison to other occupations. Wang et al. [7] in their recent study from China found students to experience a higher level of psychological distress. Besides the psychological impact of COVID-19, uncertainty and potentially negative impact on academics due to closure of schools for an indefinite time could exacerbate a negative impact on the psychological health of students. There is a need to develop web-based teaching activities and to start online portals so that the students remain engaged with studies and academics. This will also help in diverting them from maladaptive coping skills. Eighteen to 30 years of age group had significant anxiety and depressive symptoms in our study. Younger age (< 35 years) and students were found to be at potential risk for psychological impact in earlier studies [7, 22]. It is therefore anticipated that in people without any preexisting mental disorder, a considerable increase in psychological symptoms could occur, with some experiencing PTSD in due course. Evidence from China [32] during the current pandemic has found that this possibility of a surge in psychological disorders has been under-recognized there. Besides, those with preexisting psychological disorders will be at an increased risk of infection with the virus, will face problems in accessing facilities for testing and treatment, and will have a heightened risk of negative psychological impact during this pandemic [23]. Similarly, there are reports of severe negative psychological impact in frontline health care workers in comparison to those in secondary roles [33]. Emotional health is a well-recognized public health priority in disasters and it is vital to build resilience in the general public toward the reduction of the negative mental health impact of the disasters [34]. The significance of concern for mental health problems in this pandemic forced “Mental Health UK” [35] to issue psychological first aid guidance. WHO also gave a good consideration for mental health and published “Mental health and psychosocial considerations during the COVID-19 outbreak” on its website [13]. After the Middle East respiratory syndrome (MERS), SARS, and the Influenza outbreak, there were some changes made in policies, but in India, preparedness for mental and psychosocial health during a pandemic has mostly been on the backseat [36, 37]. To mitigate the negative impact of this Covid-19 pandemic on psychological health, several measures can be taken. First, it might appear attractive to deploy professionals from mental health to other areas of healthcare during this pandemic; this should not happen. There is already a shortage of mental health professionals to cater to the psychiatric needs of our population and such a move to re-deploy professionals from mental health would worsen overall outcomes, besides placing persons with mental disorders at increased risk of deteriorations in their physical as well as mental health [38]. Second, we recommend issuing guidelines for psychological help just as provided by the UK for the mental well-being of people. These measures can help to prevent or to minimize future psychiatric morbidity. Finally, a special focus is needed for the psychological well-being of people working as frontline healthcare staff.

Poor quality of sleep was present in around half of our respondents. In most of the cases with this poor quality of sleep, there was difficulty in falling asleep each night, there were more early morning awakenings, most of them had a total sleep of less than 6 hours, and there were frequent night awakenings. Some of them with poor quality of sleep took medicine to get a sound sleep while others had bad dreams. All of them rated their quality of sleep either as fairly bad or as very bad. Significant higher mean scores on PSQI were present in females. The global score on PSQI was also correlated positively with both anxiety and depressive symptoms’ scores. Roy et al. [20] however reported 28% of the participants had sleeping difficulty and 12% reported this difficulty because of being apprehensive in the past week about the pandemic. In a study by Chakraborty and Chatterjee [21], near about one third of their respondents reported disturbed sleep-wake cycle in the past 2 weeks. A higher proportion of respondents with sleep problems in our study could be because we assessed sleep for the past month. Our results are consistent with a study by Cellini et al. [39] who reported poor quality of sleep in a high proportion of their study group. Huang and Zhao [22], however, in their web-based survey found poor sleep quality only in 18% of their respondents. Women are more likely to have sleep problems in comparison to men [40]. Poor quality of sleep is a common problem related to stress [41]. Further, people sensitive to stress-induced problems in sleep are at an increased risk to develop chronic insomnia [42, 43]. Liu et al. [44] reported better quality of sleep with lesser early morning awakenings in those with less PTSD-related symptoms during this pandemic. A task force of the European Academy for Cognitive-Behavioral Treatment of Insomnia (European CBT-I Academy) [41] stated that during the lockdown period in the COVID-19 outbreak, there are various factors which challenge the sleep habits of individuals including psychological distress, reduced exposure to sunlight, and reduced physical activity. Sleep plays an important role to regulate emotions, and a problematic sleep at night can have adverse consequences on the next day’s emotional functioning [41]. Taskforce for European CBT-I Academy published practical recommendations [41] to deal with problems related to sleep during confinement at home for various target groups like the general public, women, children, and healthcare staff.

Around one third of our respondents were indulged in maladaptive coping skills to deal with ongoing stress, e.g., some of respondents started smoking while others increased frequency of smoking due to the stress during this pandemic. Few others were engaged in excessive washing and bathing, even when they were staying at home. Few others were excessively watching news related to coronavirus on television or social networking sites. There were many other such responses which were classified as maladaptive. Those who used maladaptive skills had significantly higher mean ranks for scores on anxiety symptoms, depressive symptoms, and PSQI. Adaptive coping skills are more effective in dealing with difficult situations and in managing negative emotions than maladaptive skills [45]. It is of paramount importance to understand the effective, individualized ways of coping in a situation like this pandemic [46]. The social and personal resources (e.g., proper balanced diet, proper sleep, physical exercise, and social communication at home with loved ones and with other family members and friends by email and phone) available with us can act as important resilience-related factors for minimizing psychological impact and mental health difficulties under stressful conditions like this pandemic [47].

### Limitations

It was a cross-sectional study that limited us to analyze our respondents over a period of time. This also limited us to determine the cause and effect relationship. We used social networking sites to deliver our questionnaire; this gives the possibility of selection bias. The questionnaire was presented in the English language, so only educated people could respond. Further, non-probability sampling has inherent disadvantages like lack of representation of the reference population, difficulties in estimating variability in sampling, and identifying a possible bias lower level of generalization of findings compared to probability sampling. Besides, the influence of geopolitical scenarios in our part of the world can act as a strong confounding factor.

## Conclusion

Despite these limitations, during the current extraordinary circumstances, it is expected to have a rise in anxiety and depressive symptoms and use of different coping skills, but there is always a risk that the prevalence of people with clinically relevant anxiety and depression will increase. In our study, we found anxiety and depressive symptoms, and poor quality of sleep highly prevalent in our respondents. Younger people, females, those living in urban conditions, and those using maladaptive coping skills are at increased risk to have anxiety and depressive symptoms as well as poor quality of sleep. Because the pandemic is likely to continue, there is an urgent need to monitor the psychological health of the population, besides their physical health, and to develop strategies based on evidence to minimize adverse impact on psychological health caused by these unprecedented and extraordinary changes in the daily lives of people.

## Data Availability

The data generated or analyzed during this survey are available from the corresponding author on reasonable request.

## Abbreviations

COVID-19: The 2019 coronavirus disease
SARS: Severe acute respiratory syndrome
WHO: World Health Organization
HADS: Hospital Anxiety and Depression Scale
PSQI: Pittsburgh Sleep Quality Index
SPSS: Statistical Package for Social Sciences
PTSD: Posttraumatic stress disorder
MERS: Middle East respiratory syndrome
European CBT-I Academy: European Academy for Cognitive-Behavioral Treatment of Insomnia
